# Supplementary material for: The interactome of intact mitochondria by cross-linking mass spectrometry provides evidence for coexisting respiratory supercomplexes
Source: Mol Cell Proteomics. 2017 Dec 8;17(2):216–32. doi: 10.1074/mcp.RA117.000470 (PMC5795388; doi:10.1074/mcp.RA117.000470)
Supplement: Supplemental Data [file supp_RA117.000470_133922_0_supp_23978_fzffwf.zip › spectra_annotation/mito_DR_spectra_annotation/362-1-10-1-5-1.pdf]

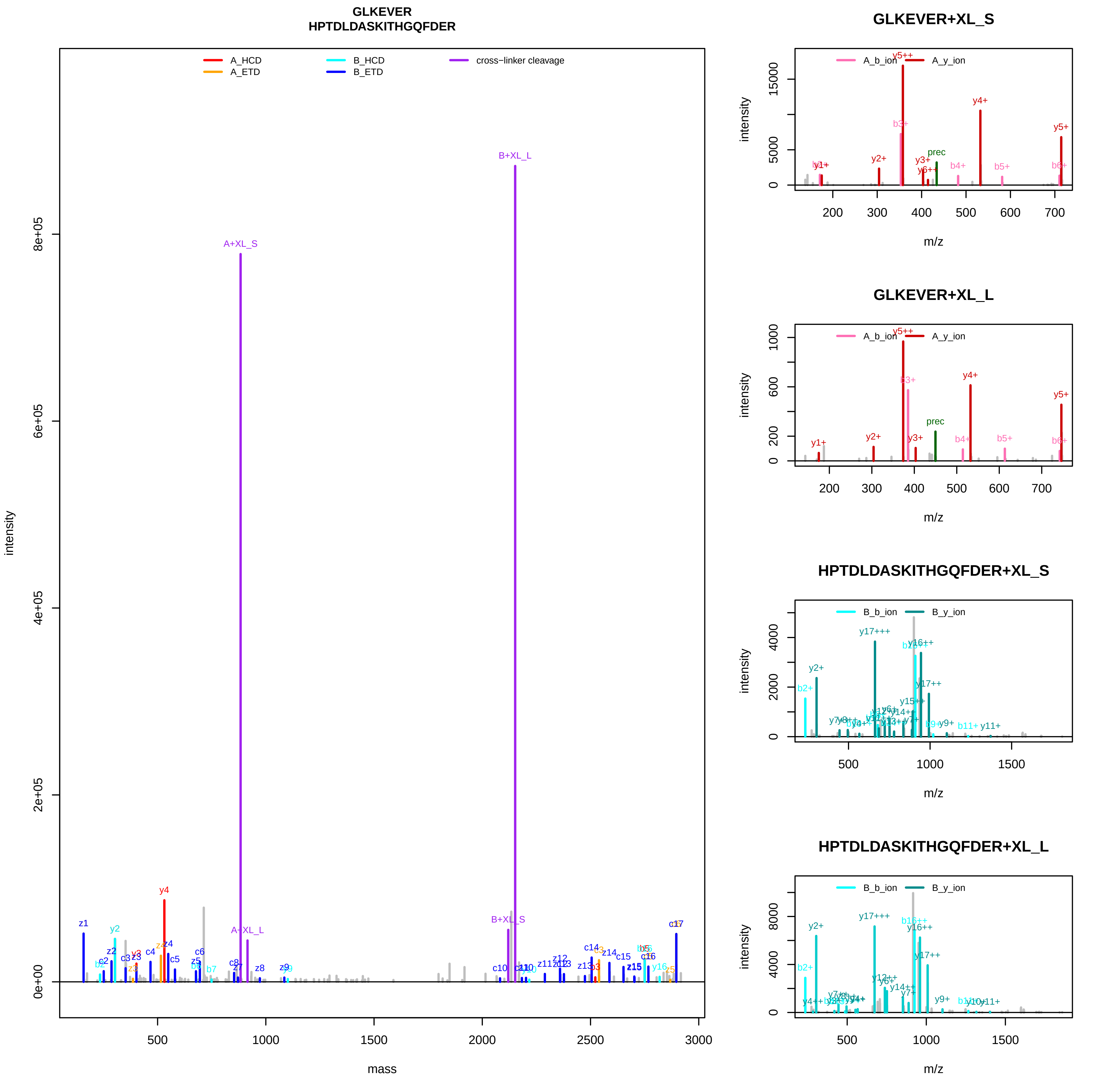
GLKEVER
HPTDLDASKITHGQFDER
A_HCD
A_ETD
B_HCD
B_ETD
cross−linker cleavage
intensity
mass
A+XL_S
B+XL_L
A+XL_L
B+XL_S
y4
z1
y2
c17
GLKEVER+XL_S
GLKEVER+XL_L
HPTDLDASKITHGQFDER+XL_S
HPTDLDASKITHGQFDER+XL_L
A_b_ion
A_y_ion
B_b_ion
B_y_ion
y5++
y4+
y5+
b3+
y2+
y1+
prec
b4+
b5+
b6+
y17+++
y16++
y17++
y15++
b2+
m/z
